# Supplementary material for: Identification of photoperiod-induced specific miRNAs in the adrenal glands of Sunite sheep (Ovis aries)
Source: Front Vet Sci. 2022 Jul 22;9:888207. doi: 10.3389/fvets.2022.888207 (PMC9354845; doi:10.3389/fvets.2022.888207)
Supplement: Supplementary file 1 [file Data_Sheet_1.ZIP › Supplementary file/miRDeep2 file.zip!/miRDeep2 file/Know_Structure/SP42A2/oar-mir-22.pdf]

miRBase precursor : oar-mir-22
Total read count : 145267
oar-miR-22-3p read count : 135476
remaining reads : 9791

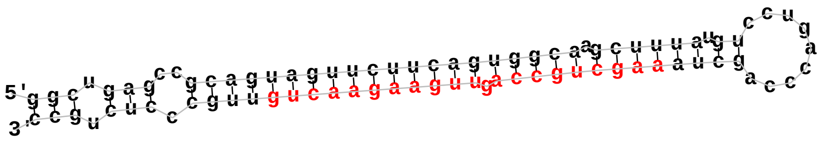

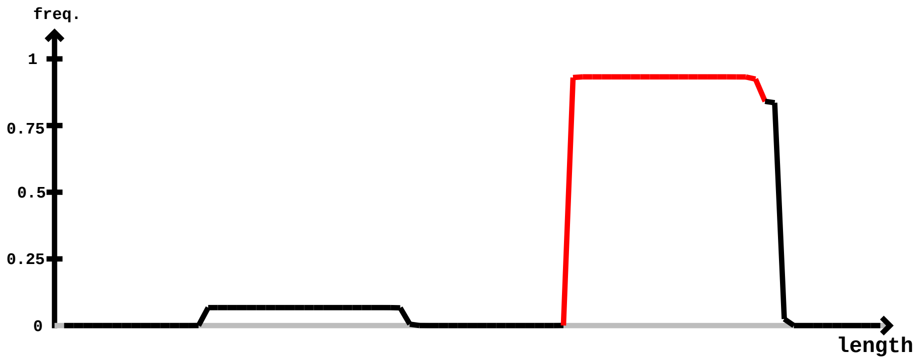

oar-miR-22-3p

```
5'- ggcugagccgcaguaguucuucaguggcaagcuuuauguccugacccagcuaaagcugccaguugaagaacuguugcccucugcc  -3'    exp
    (((.(((..((((((((((((((((((((.((((((.((.........)))))))))))))).))))))))))))))).))).)))    reads      mm         sample
    .............uaguucuucaguggcaagcuu...................................................    2          0          seq
    .............uaguucuucaguggcaagcuuu..................................................    23         0          seq
    .............uaguucuucaguggcaagcuuua.................................................    2          0          seq
    ..............aguucuucaguggcaagc.....................................................    3          0          seq
    ..............aguucuucaguggcaagU.....................................................    1          1          seq
    ..............aguucuucaguggcaagcu....................................................    7          0          seq
    ..............aguucuucaguggcaagcuu...................................................    114        0          seq
    ..............aguucuucaguggcaagcuuu..................................................    8865       0          seq
    ..............aguucuucaguggcaagcuuua.................................................    730        0          seq
    ..............aguucuucaguggcaagcuuuau................................................    32         0          seq
    ...............guucuucaguggcaagcuuu..................................................    8          0          seq
    ...............guucuucaguggcaagcuuua.................................................    1          0          seq
    .................ucuucaguggcaagcuuuauguc.............................................    1          0          seq
    ............................................cccagcuaaagcugccaguugaagaacu.............    1          0          seq
    .................................................cuaaagcugccaguugaagaacu.............    1          0          seq
    ..................................................uaaagcugccaguugaagaacugu...........    1          0          seq
    ...................................................aaagcugccaguugaagaacu.............    20         0          seq
    ...................................................aaagcugccaguugaagaacugu...........    21         0          seq
    ....................................................aagcugccaguugaaga................    24         0          seq
    ....................................................aagcugccaguugaagaa...............    37         0          seq
    ....................................................aagcugGcaguugaagaa...............    1          1          seq
    ....................................................aagcugccaguugaagaac..............    1152       0          seq
    ....................................................aagcugccaguuCaagaacu.............    2          1          seq
    ....................................................aagcugccaguugaagaacu.............    12075      0          seq
    ....................................................aagcugccaguugaagaacug............    774        0          seq
    ....................................................aagcugccaguugaagaacugu...........    117467     0          seq
    ....................................................aagcugccaguugaagaacuguu..........    3538       0          seq
    ....................................................aagcugccaguugaagaacuguug.........    5          0          seq
    .....................................................agcugccaguugaagaacu.............    22         0          seq
    .....................................................agcugccaguugaagaacugu...........    303        0          seq
    .....................................................agcugccaguugaagaacuguu..........    27         0          seq
    ......................................................gcugccaguugaagaacu.............    1          0          seq
    ......................................................gcugccaguugaagaacugu...........    1          0          seq
    ......................................................gcugccaguugaagaacuguu..........    4          0          seq
    .......................................................cugccaguugaagaacugu...........    1          0          seq
```

oar-miR-22-3p

ggcugagccgcaguaguucuucaguggcaagcuuuauguccugacccagcuaaagcugccaguugaagaacuguugcccucugcc
